# Supplementary material for: Development of a novel startle response task in Duchenne muscular dystrophy
Source: PLoS One. 2022 Apr 19;17(4):e0264091. doi: 10.1371/journal.pone.0264091 (PMC9017900; doi:10.1371/journal.pone.0264091)
Supplement: S1 File — This table presents study protocol details obtained from nine previously published studies using fear-conditioning tasks with paediatric participants, which informed some aspects of the development of the novel task we created for this study. References: 1Gao Y, Raine A, Venables PH, Dawson ME, Mednick SA. The development of skin conductance fear conditioning in children from ages 3 to 8 years. Dev Sci. 2010;13(1):201–12.; 2Pattwell SS, Duhoux S, Hartley CA, Johnson DC, Jing D, Elliott MD, et al. Altered fear learning across development in both mouse and human. Proc Natl Acad Sci USA. 2012;109(40):16318–23.; 3Neumann DL, Waters AM, Westbury HR, Henry J. The use of an unpleasant sound unconditional stimulus in an aversive conditioning procedure with 8- to 11-year-old children. Biol Psychol. 2008;79(3):337–42.; 4Shechner T, Britton JC, Ronkin EG, Jarcho JM, Mash JA, Michalska KJ, et al. Fear conditioning and extinction in anxious and nonanxious youth and adults: examining a novel developmentally appropriate fear-conditioning task. Depress Anxiety. 2015;32(4):277–88.; 5Field AP. I don’t like it because it eats sprouts: conditioning preferences in children. Behav Res Ther. 2006;44(3):439–55.; 6Lau JY, Lissek S, Nelson EE, Lee Y, Roberson-Nay R, Poeth K, et al. Fear conditioning in adolescents with anxiety disorders: results from a novel experimental paradigm. J Am Acad Child Adolesc Psychiatry. 2008;47(1):94–102.; 7Glenn CR. Comparing electric shock and a fearful screaming face as unconditioned stimuli for fear learning. Int J Psychophysiol. 2012;86(3):214–9.; 8Jovanovic T, Nylocks KM, Gamwell KL, Smith A, Davis TA, Norrholm SD, et al. Development of fear acquisition and extinction in children: effects of age and anxiety. Neurobiol Learn Mem. 2014;113:135–42.; 9Schiele MA, Reinhard J, Reif A, Domschke K, Romanos M, Deckert J, et al. Developmental aspects of fear: Comparing the acquisition and generalization of conditioned fear in children and adults. Dev Psychobiol. 201 [file pone.0264091.s001.pdf]

	**Gao *et al*. (2010)**[1]	**Pattwell *et al*. (2012)**[2]	**Neumann *et al*. (2008)**[3]	**Shechner *et al*. (2015)**[4]	**Field *et al*. (2006)**[5]	**Lau *et al*. (2008)**[6]	**Glenn *et al*. (2012)**[7]	**Jovanovic *et al*. (2014)**[8]	**Schiele *et al*. (2016)**[9]
**Subjects**									
**Age range**	3-8 years	5 - 28 years	8-17 years	youth/adults			young adults	8-13 years	8-10; 18-50
**Total number**	200		16	youth: 37<br>adults: 47		54	40	60	children: 239<br>adults: 278
**Healthy/ pathology**	Healthy	Healthy	Healthy	Healthy/ anxious	Healthy	Healthy (38)/ anxious (16)		Low/high anxiety	Healthy
**Conditioned stimulus (CS)**									
**CS+/CS-**	1000Hz/500Hz 60dB tone	Coloured squares	Black & white squares	Coloured bell pictures	Neutral cartoons	Neutral faces	Neutral faces & shapes	Coloured shapes	Neutral faces
**Duration (s)**	12.5	3	8	8	3	8	6	0.50	6
**Unconditioned stimulus (UCS)**									
**UCS**	White noise + rattling keys	White noise + tone	Metal on slate sound	Alarm & red bell pic	Food pictures	Fearful face + scream	Fearful face + scream/shock	Noise burst	Fearful face and scream
**Duration (s)**	4.5	1	3	1	2	3	3 (face); 1 (scream)	0.04	1.5
**Onset (s)**	10	2	5	7	1	at CS+ offset	at CS+ offset	same time	at CS+ offset
**Offset (s)**	2s after CS+	with CS+	with CS+	with CS+	with CS+				
**dB level**	90	85-95 (child); 94-104 (adol/adult)	83	95	n/a	95	80	106	95
**Mean inter-trial interval (ITI) (s)**	38	13	14.5	14.5	3		11	15.5	10.5
**ITI range (s)**	34-42	13	13-16	8-21	2-4		10-12	9-22	9-12
**Analysis window**		1-10s		0-5s				3-6s	
**Trial protocol**									
**Orienting/pre-exposure**	6 neutral tones		3 min baseline	6 startle probes					
**No. Familiarisation trials**			2 CS+/2CS-	4 CS+/4 CS-		4 CS+/4 CS-		0	4 CS+/4 CS-
**No. Acquisition trials**									
**CS+ (reinforced)**	6	12	12	8	20	12	6	3	10
**CS+ (not reinforced)**	3	12	0	2	0	4	2	3	2
**CS-**	3	24	12	10	4	16	8	3	12
**No. Extinction trials:**	n/a	24 CS+/24 CS-	12 CS+/12 CS-	8 CS+/8 CS-	20 CS+	15 CS+/15 CS-		12 CS+	n/a
**Counter-balanced CS**	N	Y	Y	Y	N	N	Y	N	
